# Investigation of the midgut structure and ultrastructure in *Cimex lectularius* and *Cimex pipistrelli* (Hemiptera: Cimicidae)

**DOI:** 10.1007/s13744-016-0430-x

**Published:** 2016-08-23

**Authors:** M M Rost-Roszkowska, J Vilimova, A Włodarczyk, L Sonakowska, K Kamińska, F Kaszuba, A Marchewka, D Sadílek

**Affiliations:** 1 Dept of Animal Histology and Embryology, Univ of Silesia, Bankowa 9, 40-007 Katowice, Poland; 2 Faculty of Science, Dept of Zoology, Charles Univ, Praha 1, Czech Republic

**Keywords:** Midgut epithelium, alimentary tract, digestive cells, secretory cells

## Abstract

Cimicidae are temporary ectoparasites, which means that they cannot obtain food continuously. Both *Cimex* species examined here, *Cimex lectularius* (Linnaeus 1758) and *Cimex pipistrelli* (Jenyns 1839), can feed on a non-natal host, *C. lectularius* from humans on bats, *C. pipistrelli* on humans, but never naturally. The midgut of *C. lectularius* and *C. pipistrelli* is composed of three distinct regions—the anterior midgut (AMG), which has a sack-like shape, the long tube-shaped middle midgut (MMG), and the posterior midgut (PMG). The different ultrastructures of the AMG, MMG, and PMG in both of the species examined suggest that these regions must fulfill different functions in the digestive system. Ultrastructural analysis showed that the AMG fulfills the role of storing food and synthesizing and secreting enzymes, while the MMG is the main organ for the synthesis of enzymes, secretion, and the storage of the reserve material. Additionally, both regions, the AMG and MMG, are involved in water absorption in the digestive system of both *Cimex* species. The PMG is the part of the midgut in which spherites accumulate. The results of our studies confirm the suggestion of former authors that the structure of the digestive tract of insects is not attributed solely to diet but to the basic adaptation of an ancestor.

## Introduction

The digestive system of insects is composed of three distinct regions—the ectodermal foregut and hindgut and the endodermal midgut, which lies between the ectodermal regions. The foregut and hindgut are lined with the cuticle, while the midgut is devoid of this structure. The shape and length of the midgut, which depend on the type of food that the animal eats (Billingsley 1990, Silva *et al* 1995, Chapman 1998), can be differentiated—it can have many caeca that are located anteriorly or/and posteriorly or it can be differentiated into distinct regions: the anterior midgut, middle midgut, and posterior midgut. However, the midgut is more commonly tube-shaped (Billingsley 1990, Ponsen 1991, Silva *et al* 1995, Rost-Roszkowska 2008). The more complicated structure can be observed in insects that feed on fluids, e.g., plant/animal fluids or blood (Billingsley 1990, Ponsen 1991, Le Caherec *et al* 1997).

From Insecta, only the Hemiptera have a unique plant sap-sucking capability, which has caused specific modifications of their alimentary tract to facilitate the absorption of nutrients that have very low concentrations in phloem or xylem sap. The results of these modifications were the loss of the peritrophic membrane in a Hemiptera ancestor in order to adapt to sucking phloem, the development of lipoprotein perimicrovillar membranes (absorption of amino acids), and the loss of serine proteases (feeding on sap that is devoid of macromolecules). However, lysosomal protease is used in sap-sucking Heteroptera that have returned to a protein-rich feeding environment (as for example blood) (Silva *et al* 1995).

The successful adaptive radiation of the Hemiptera, including Heteroptera, is thought to be based on the wide range of feeding habitats that are facilitated by highly modified piercing and sucking mouthparts. Although Hemiptera originally fed on plant sap, the Heteroptera especially began to also feed on seeds, the tissues of other animals (zoophagy) and the body fluids of animals (parasitism) including the blood of vertebrates (Goodchild 1966). Together with Cimicidae, also Polyctenidae and Reduviidae (Triatominae) feed on blood, whereas triatomine *Rhodnius prolixus Stal* has been studied as model blood sucking species (e.g., Terra 1988, 1990, Billingsley 1990).

The *Cimex* species are obligatory blood feeders; blood serves as the sole source for ingested nutrients and water. Thus, the hematophagy requires specific enzymes and associated pathway to digest efficiently blood (Benoit *et al* 2007). Evolutionary adaptations including expansion of genes that are associated with blood digestion were recognized during sequencing of the *Cimex lectularius* (Linnaeus) genome (Benoit *et al* 2016). The family Cimicidae (Hemiptera: Heteroptera) constitutes a group of specialized obligate hematophagous ectoparasitic insects. Cimicidae contains about 110 species in 24 genera, which are distributed world-wide (Usinger 1966, Henry 2009). These insects stay on the body of their hosts, strictly birds and mammals, only when feeding. Most of the cimicids are associated primarily with bats, e.g., *Cimex pipistrelli* Jenyns, which are the suggested original host of the family (Horváth 1913). Three bat-associated species, including *C. lectularius* have developed continuous populations that are parasitic to humans (e.g., Usinger 1966, Balvín *et al* 2012, Booth *et al* 2015). *C. lectularius* was practically eradicated in developed countries in the last century due to the widespread use of DDT; however, recently, it has undergone a global spread due to increased international travel, global commerce, a resistance to pyrethroid insecticides, etc. (Reinhardt & Siva-Jothy 2007, Romero *et al* 2007, Reinhardt *et al* 2008, Davies *et al* 2012, Wang *et al* 2013, Lilly *et al* 2015). This species is currently being studied intensively due to its potential to be a medically important vector. To date, there have been no reports of natural pathogen transmission by *C. lectularius* to humans; however, numerous pathogen microorganisms and viruses can survive in that species in laboratory conditions (Zorrilla-Vaca *et al* 2015), e.g., *C. lectularius* is a vector of *Trypanosoma cruzi* (Salazar *et al* 2015). *Cimex pipistrelli* is the common exclusively bat-associated European *Cimex* species (Balvín *et al* 2013, 2014). Both *Cimex* species are acyclic and pass through five nymphal instars during ontogeny. The temperature for hatching, nymphal development (five nymphal instars) and adult activity is 13–15°C. Eggs are produced after mating and are laid individually in and around harbourages of hosts. Temperature and frequency of food intake affect all life processes.

The first morphological description of the digestive tract of *C. lectularius* was published by Miyamoto (1961) and then by Forattini (1990); however, no ultrastructure of the midgut of any *Cimex* species was recognized until the study of *Cimex hemipterus* (Fabricius, 1803) by Azevedo *et al* (2009). *Cimex lectularius* and *C. pipistrelli*, including their digestive tracts, are currently being studied in detail in Central Europe on a large sample of rare material of both species, which is difficult to collect. The aim of this paper is to study the midgut ultrastructure of hematophagous species in which there were long intervals of starvation between blood meals and to compare two species from different hosts.

## Material and methods

### Material

Several dozens of *C. lectularius* specimens of both sexes from human hosts were studied. Samples were collected from human dwellings from April 2013 until March 2015 with the assistance of pest exterminator specialists. The *C. lectularius* material that was studied originated from six localities in the Czech Republic, one locality from Poland, and one from France. Several dozens of *C. pipistrelli* specimens from bat hosts were collected with the assistance of chiropterologists from September 2008 until March 2015 from four localities in the Czech Republic. The collected living *Cimex* specimens were kept at a temperature of 4°C and successively killed and fixed in different stages of food digestion, in intervals of couple of days to maximally about 3 weeks after feeding.

### Methods

The midguts, which were dissected from adult specimens of *C. lectularius* and *C. pipistrelli,* were initially fixed with 2.5% glutaraldehyde in a 0.1 M sodium phosphate buffer (pH 7.4) for 2 h. After washing in a sodium phosphate buffer, the material was postfixed for 2 h in 1% OsO_4_ in the same buffer (2 h, 4°C), dehydrated in a graded series of ethanol (50%, 70%, 90%, 96%, and 100%, 15 min each) and acetone (2 × 15 min) and then embedded in an Epoxy Embedding Medium Kit (Sigma, St. Louis, MO). Semi-thin sections (0.8 μm thick) stained with methylene blue were examined under an Olympus BX60 microscope equipped with a DP12 digital camera and AnaliSIS 3.2 (Soft Imaging System) software. Ultra-thin sections (80 nm) were cut on a Leica ultracut UCT ultramicrotome. After staining the material with uranyl acetate and lead citrate, the sections were examined using a Hitachi H500 transmission electron microscope at 75 kV.

The isolated midguts from two specimens of *C. lectularius* and two specimens of *C. pipistrelli* were dissected and photographed using an Olympus SZ-ST stereomicroscope.

#### Detections of lipids (Sudan black B staining).

Semi-thin sections were stained with Sudan black B at room temperature (15 min) (Litwin 1985). After a quick wash with 50% ethanol and distilled water, the material was examined using an Olympus BX60 light microscope.

#### Detection of glycogen and polysaccharides (PAS method).

Semi-thin sections of the glands were treated with a 2% solution of periodic acid (10 min, room temperature) and washed in 70% ethanol and stained with Schiff’s reagent (24 h, 37°C) (Litwin 1985). After washing in water, the slides were analyzed using an Olympus BX60 light microscope.

#### Detection of proteins (Bonhag method).

Semi-thin sections were treated with a 1% solution of periodic acid (10 min, room temperature), washed in water, and stained with bromophenol blue (BPB) (24 h, 37°C) (Litwin 1985). After washing the slides with water, they were analyzed using an Olympus BX60 light microscope.

## Results

**Fig 1:**
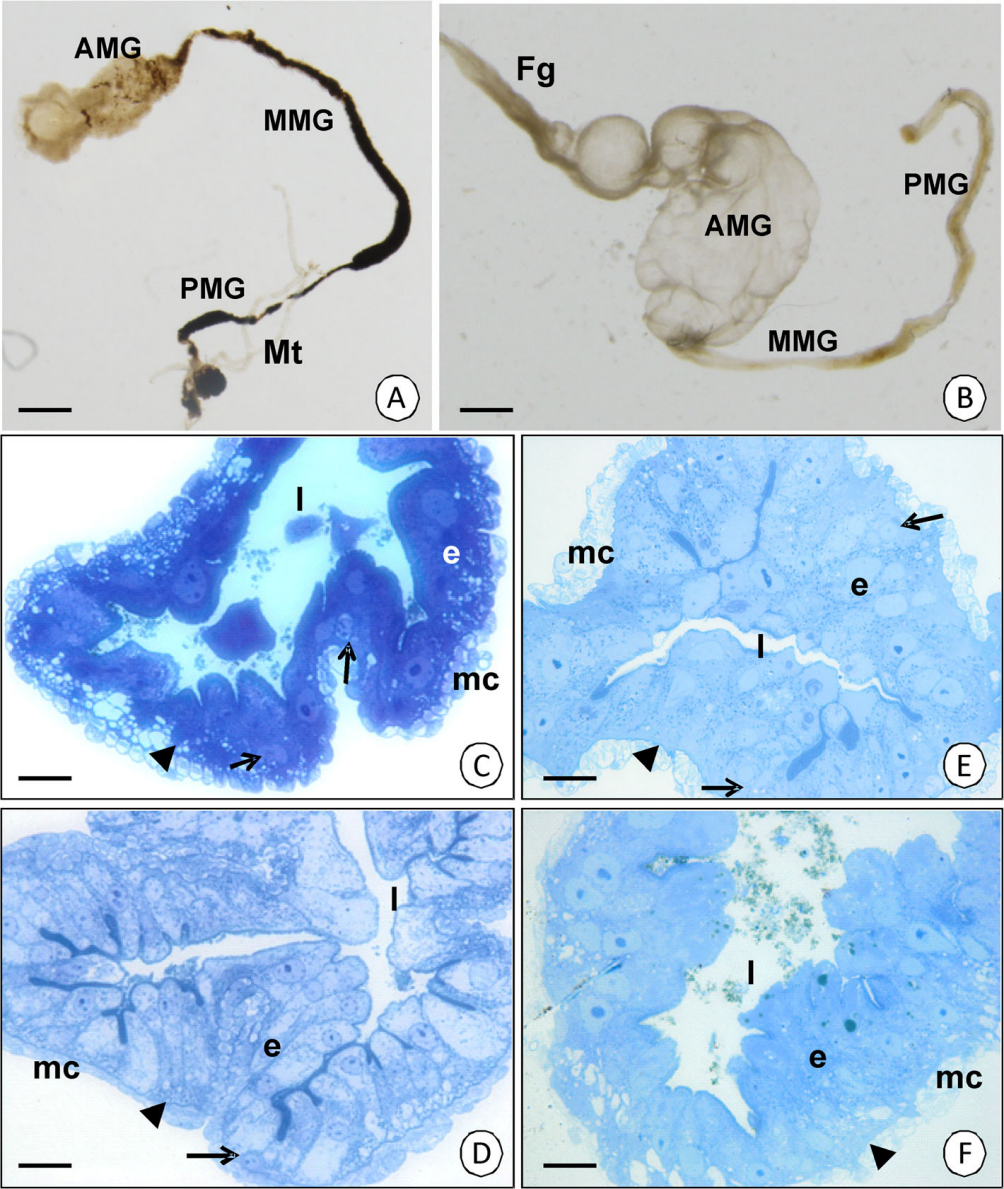
Midgut of *Cimex lectularius* (**A**) and *Cimex pipistrelli* (**B**) divided into anterior (AMG), middle (MMG), and posterior midgut (PMG). *Fg* fragment of the foregut, Malpighian tubules. **A** Stereomicroscope. *Bar* = 2.7 mm. **B** Stereomicroscope. *Bar* = 1.5 mm. **C** *Cimex lectularius*. The transverse section through AMG. Light microscope. *Bar* = 5 μm. **D** *Cimex lectularius.* The transverse section through MMG. Light microscope. *Bar* = 4 μm. **E** *Cimex pipistrelli.* The transverse section through PMG. Light microscope. *Bar* = 4 μm. **F** *Cimex pipistrelli.* The transverse section through MMG. Light microscope. *Bar* = 4 μm. Midgut lumen (*l*), midgut epithelium (*e*), visceral muscles (*mc*), basal lamina (*arrowhead*), regenerative cells (*arrows*).

The midgut of *C. lectularius* and *C. pipistrelli* is composed of three distinct regions—the anterior midgut (AMG), which has a sack-like shape, the long tube-shaped middle midgut (MMG), and the posterior midgut (PMG) (Fig 1A, B). In *C. lectularius*, the MMG forms about 50% of the whole midgut length, while the AMG and PMG form only about 25% each. However, the AMG and MMG in *C. pipistrelli* constitute about 25% each, while the PMG, which is the longest part of the midgut, covers about 50% of the midgut length. They are formed by a simple columnar (AMG and MMG) or simple cuboidal (PMG) epithelium that lies on the non-cellular basal lamina and is surrounded by the visceral muscles (Fig 1C–F). Three types of cells, digestive cells, secretory cells, and regenerative cells, are present in the midgut epithelium in all three regions (Fig 1C–E).

### Digestive cells

**Fig 2:**
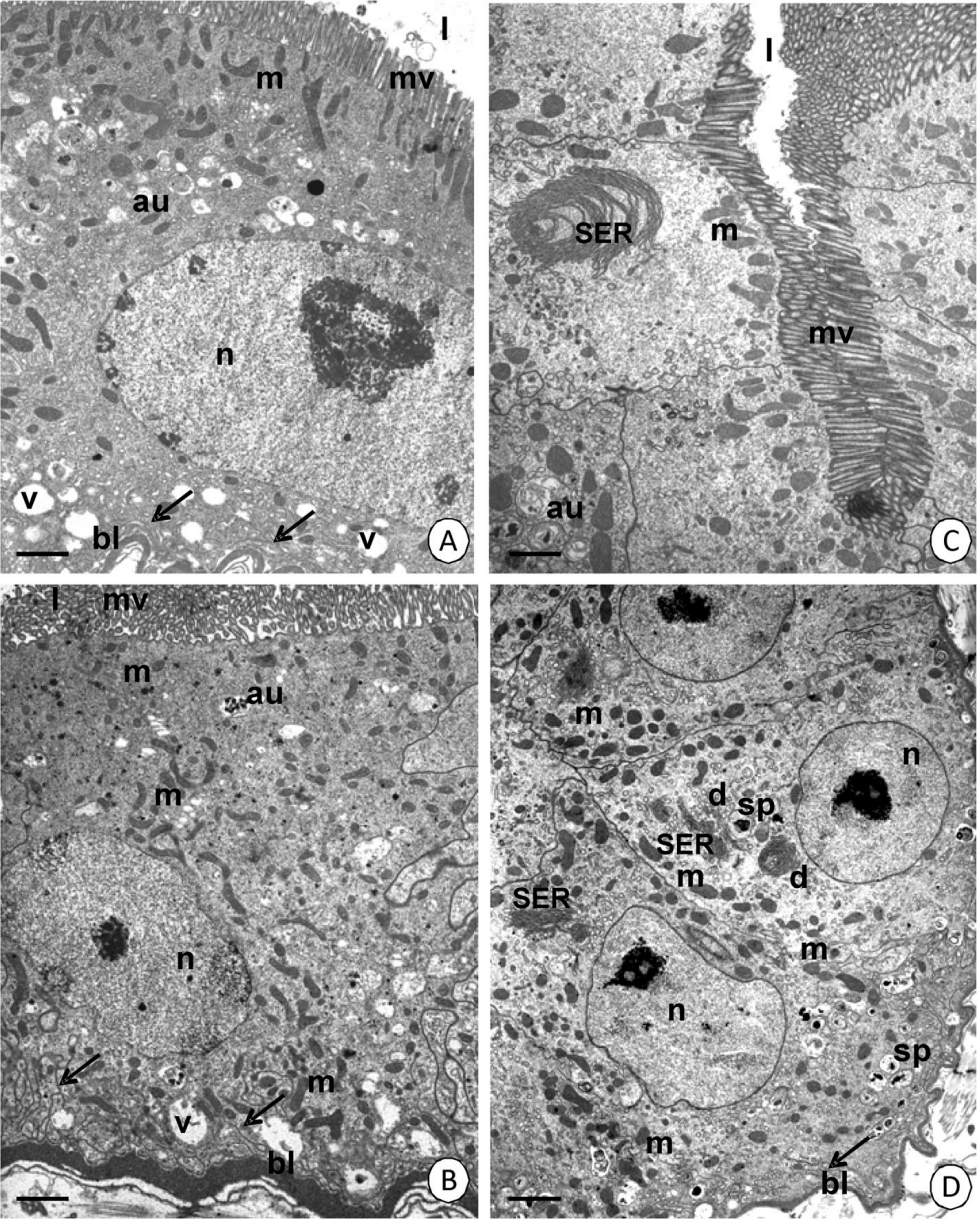
Digestive cells in the epithelium of three regions of the midgut. **A** *Cimex lectularius*. AMG. TEM. *Bar* = 0.7 μm. **B** *Cimex lectularius*. MMG. TEM. *Bar* = 1 μm. **C** *C. pipistrelli*. MMG. TEM. *Bar* = 1 μm. **D** *Cimex pipistrelli*. PMG. TEM. *Bar* = 1.4 μm. Midgut lumen (*l*), microvilli (*mv*), mitochondria (*m*), nucleus (*n*), basal lamina (*bl*), basal cell membrane folds (*arrows*), autophagosomes (*au*), spherites (*sp*), cisterns of the smooth endoplasmic reticulum (*SER*), Golgi complexes (*d*), vacuoles (*v*).

**Fig 3:**
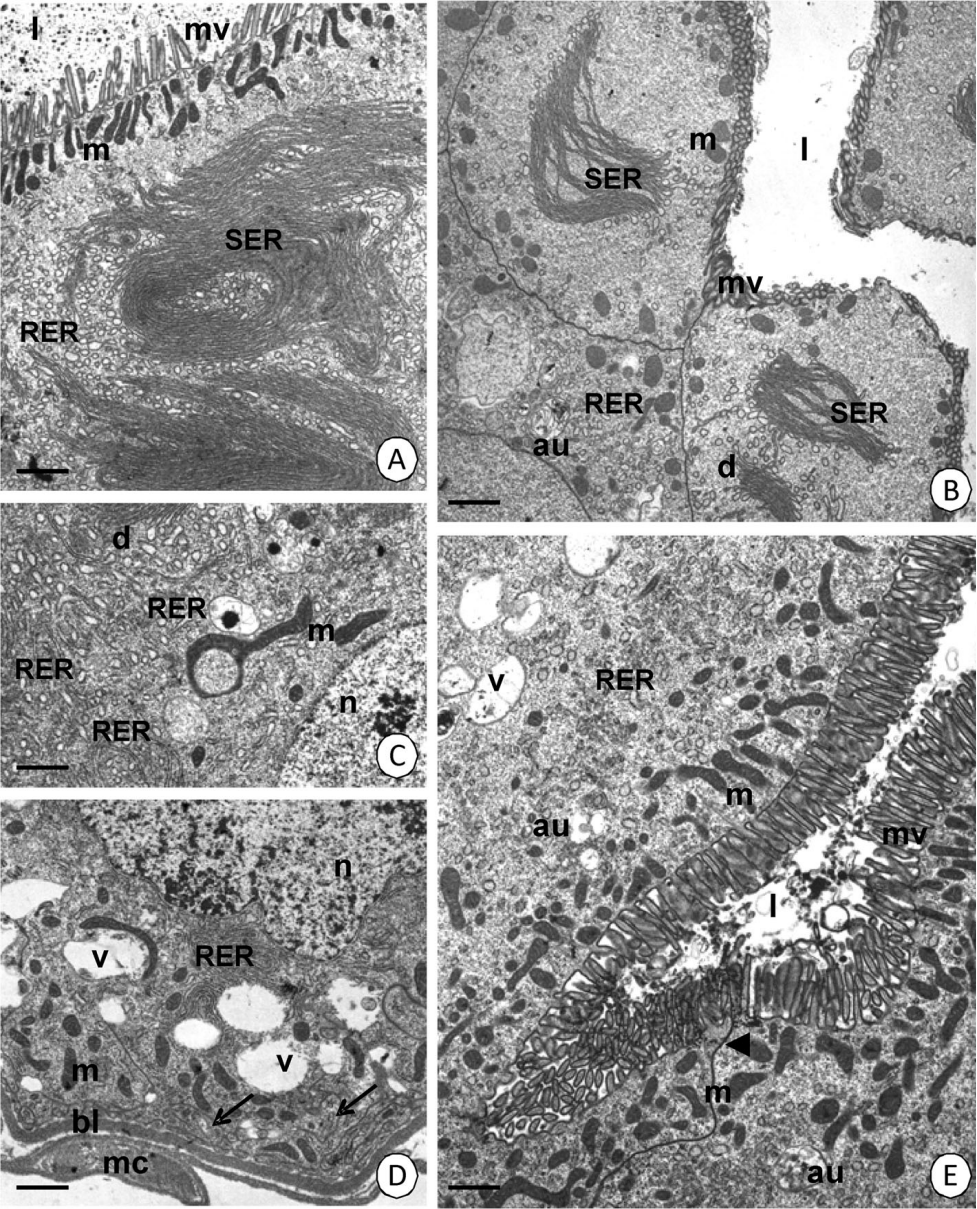
The ultrastructure of the midgut epithelium just before eating. **A** *Cimex lectularius*. MMG. TEM. *Bar* = 0.7 μm. **B** *Cimex pipistrelli*. MMG. TEM. *Bar* = 1 μm. **C** *C. lectularius*. AMG. TEM. *Bar* = 0.4 μm. **D** *Cimex lectularius*. AMG. TEM. *Bar* = 0.7 μm. **E** *C. pipistrelli*. PMG. TEM. *Bar* = 0.8 μm. Midgut lumen (*l*), mitochondria (*m*), microvilli (*mv*), autophagosomes (*au*), cisterns of the rough (*RER*) and smooth (*SER*) endoplasmic reticulum, Golgi complexes (*d*), nucleus (*n*), vacuoles (*v*), smooth septate junction (*arrowhead*), folds of the basal cell membrane (*arrows*), basal lamina (*bl*), visceral muscles (*mc*).

The columnar cells (in AMG and MMG) and cuboidal cells (in PMG) are the principal cells of the entire midgut epithelium. Their cytoplasm in AMG and MMG in both species shows a similar ultrastructure, although there are some differences, which are described below. A distinct regionalization causes the appearance of the basal, perinuclear and apical cytoplasm (Fig 2A–C). When the midgut lumen is devoid of food masses (blood), the apical cytoplasm shows a distinct layer of mitochondria that rests just beneath the apical cell membrane (Fig 2A, C, and 3A, B). Small and single autophagosomes can occasionally be observed in the apical cytoplasm (Fig 2A–C). Additionally, numerous large and flattened cisterns of the smooth endoplasmic reticulum (SER), which form giant fibrillar structures, appear in the apical cytoplasm of MMG. They are escorted by some small cisterns of the rough endoplasmic reticulum (Fig 3A, B). Numerous cisterns of the rough endoplasmic reticulum and some mitochondria accumulate around the nucleus in both regions, the AMG and MMG (Fig 3C). The basal cell membrane folds slightly and small vacuoles with an electron-lucent content gather in its vicinity (Fig 2B and 3D). They are accompanied by some cisterns of the rough endoplasmic reticulum and mitochondria (Fig 3D). Mitochondria accumulate just beneath the apical cell membrane, together with spherites (Fig 2D) and some autophagosomes (Fig 3E) in the apical cytoplasm of the digestive cells in PMG. The basal cell membrane folds slightly. However, it is only accompanied by mitochondria and occasionally cisterns of the rough endoplasmic reticulum; however, no vacuoles, which appear in the AMG and MMG, occur (Fig 2D). The entire cytoplasm is rich in cisterns of the smooth endoplasmic reticulum and Golgi complexes, while cisterns of the rough endoplasmic reticulum are rather scarce (Fig 2D).

**Fig 4:**
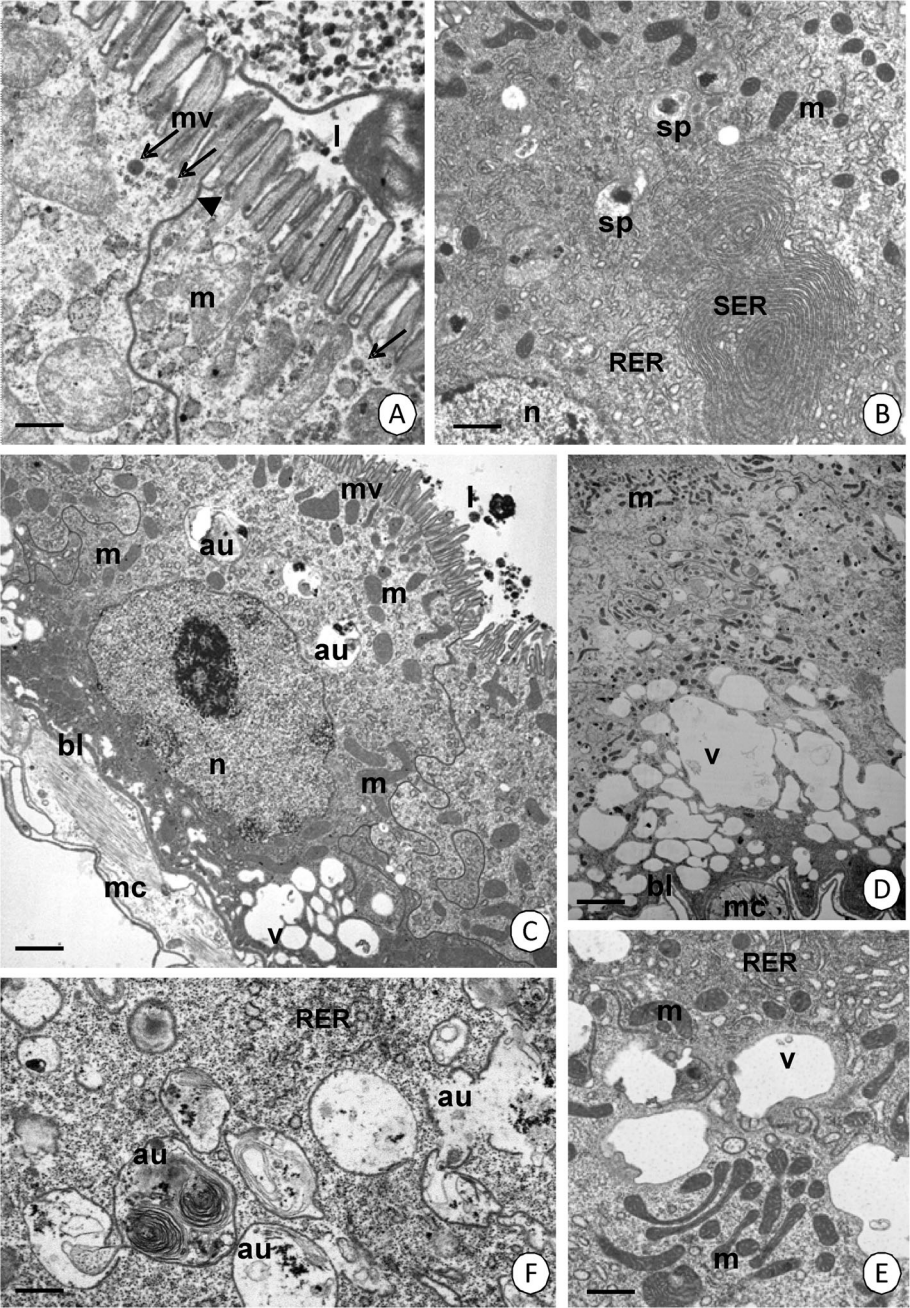
Ultrastructure of the midgut epithelium during digestion. **A** *Cimex lectularius*. MMG. Apical surface of digestive cells with vesicles with medium electron-dense content (*arrows*). TEM. *Bar* = 0.2 μm. **B** *Cimex lectularius*. AMG. TEM. *Bar* = 0.5 μm. **C** *Cimex lectularius*. MMG. TEM. *Bar* = 0.5 μm. **D** *Cimex lectularius*. AMG. TEM. *Bar* = 1.5 μm. **E** *Cimex pipistrelli*. MMG. TEM. *Bar* = 0.5 μm. **F** *Cimex pipistrelli*. MMG. TEM. *Bar* = 0.2 μm. Basal lamina (*bl*), mitochondria (*m*), microvilli (*mv*), autophagosomes (*au*), nucleus (*n*), vacuoles (*v*), midgut lumen (*l*), cisterns of the smooth (*SER*) and rough (*RER*) endoplasmic reticulum, visceral muscles (*mc*), spherites (*sp*), smooth septate junction (*arrowhead*), vesicles with electron-medium content (*arrows*).

Smooth septate junctions are present along the entire length of the midgut between neighboring digestive cells in their apical regions (Figs 3E and 4A), while septate junctions occur in the perinuclear and basal regions.

Immediately after blood sucking, when the blood enters the midgut lumen, numerous granules with a content that has a medium electron density (Fig 4A) gradually appear in the apical cytoplasm of the digestive cells in the AMG and MMG. The number of large fibrillar structures formed by cisterns of the smooth endoplasmic reticulum and Golgi complexes in the MMG gradually decreases (Fig 4B) and eventually, no fibrillar structures can be observed (Fig 4D). Together with the blood digestion, the fusion of small vesicles with an electron-lucent content that are present in the basal cytoplasm occurs and some autophagosomes occur in the majority of the digestive cells of the AMG and MMG (Fig 4C). Eventually, large vacuoles with an electron-lucent content completely fill the entire basal cytoplasm (Fig 4D). Large vacuoles are accompanied by abundant but small cisterns of the rough endoplasmic reticulum and mitochondria (Fig 4E). Occasionally, cisterns of the rough endoplasmic reticulum and numerous autophagosomes appear in the apical cytoplasm in the digestive cells in both regions, the AMG and MMG (Fig 4F).

**Fig 5:**
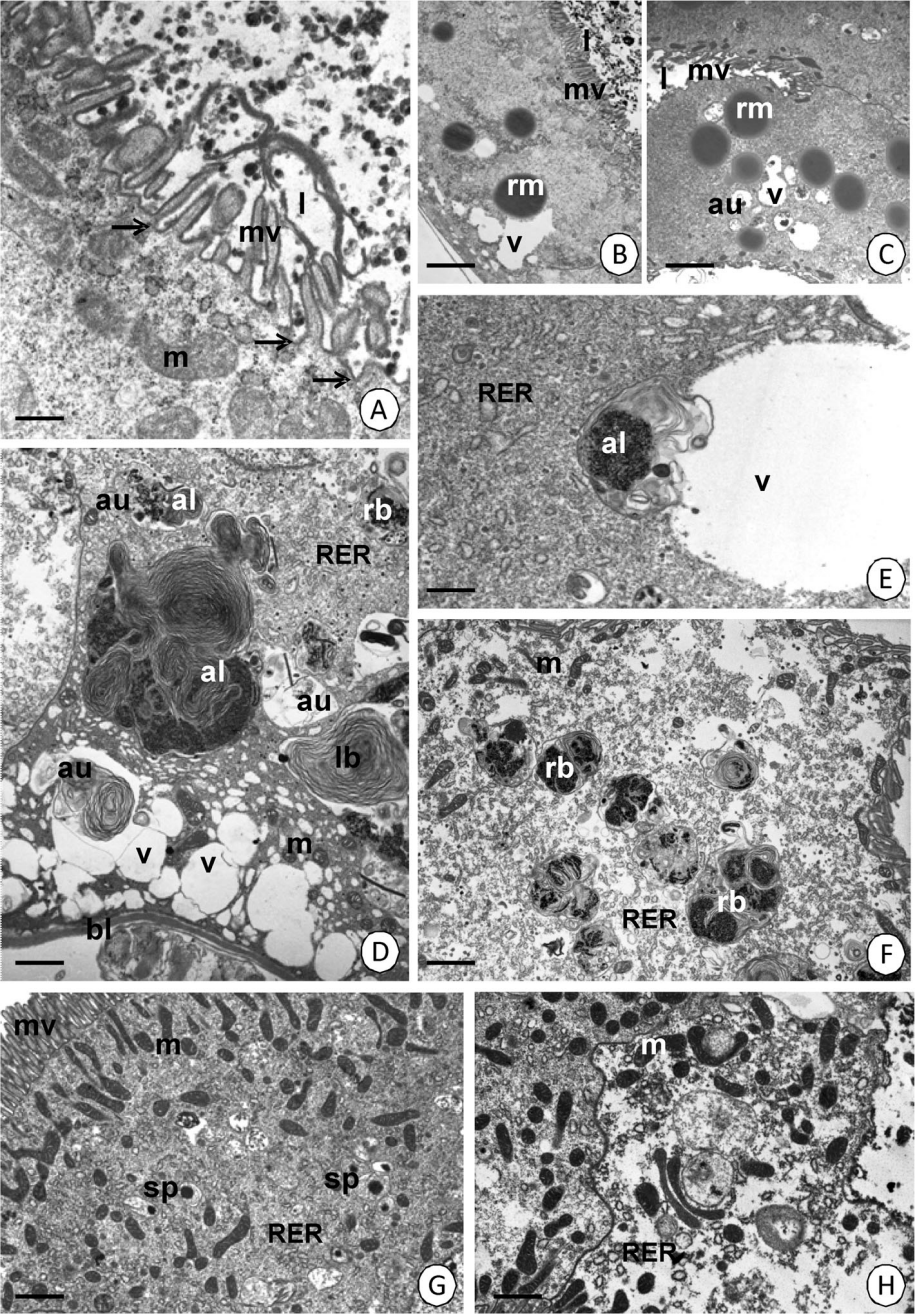
Ultrastructure of the midgut epithelium during digestion. **A** *Cimex lectularius.* MMG. TEM. *Bar* = 0.2 μm. **B** *Cimex lectularius*. MMG. TEM. *Bar* = 2.5 μm. **C** *Cimex pipistrelli*. MMG. TEM. *Bar* = 2.9 μm. **D** *Cimex pipistrelli*. MMG. TEM. *Bar* = 0.5 μm. **E** *Cimex pipistrelli.* MMG. TEM. *Bar* = 0.3 μm. **F** *Cimex pipistrelli*. MMG. TEM. *Bar* = 1.1 μm. **G** *Cimex lectularius*. PMG. TEM. *Bar* = 0.6 μm. **H** *Cimex lectularius*. PMG. TEM. *Bar* = 0.4 μm. Autophagosomes (*au*), autolysosomes (*al*), lamellar bodies (*lb*), residual bodies (*rb*), mitochondria (*m*), microvilli (*mv*), vacuoles (*v*), cisterns of the rough endoplasmic reticulum (*RER*), reserve material (*rm*), midgut lumen (*l*), exocytosis (*arrows*), basal lamina (*bl*), spherites (*sp*).

Together with blood digestion, exocytosis occurs in the digestive cells of the MMG (Fig 5A). Additionally, the reserve material accumulates in these cells (Fig 5B, C). The MMG is the only region of the midgut in which the reserve material accumulates. Simultaneously, more and more autophagosomes, autolysosomes, lamellar bodies, and residual bodies appear (Fig 5D) and some of them fuse with the vacuoles of the basal region (Fig 5E). When the cytoplasm of the digestive cell is rich in vacuoles with an electron-lucent content and autophagosomes inside, necrosis is activated. Numerous residual bodies appear. The number of organelles decreases gradually and the cytoplasm becomes electron lucent (Fig 5F). The apical cell membrane breaks and the remaining organelles are discharged into the midgut lumen (not shown). However, in the cytoplasm of the digestive cells in the PMG, the reserve material, vacuoles, and numerous vesicles with an electron-lucent content do not accumulate, although abundant spherites gather in the entire cytoplasm according to the blood digestion (Fig 5G). The digestive cells in PMG also die in a necrotic manner (Fig 5H).

**Fig 6:**
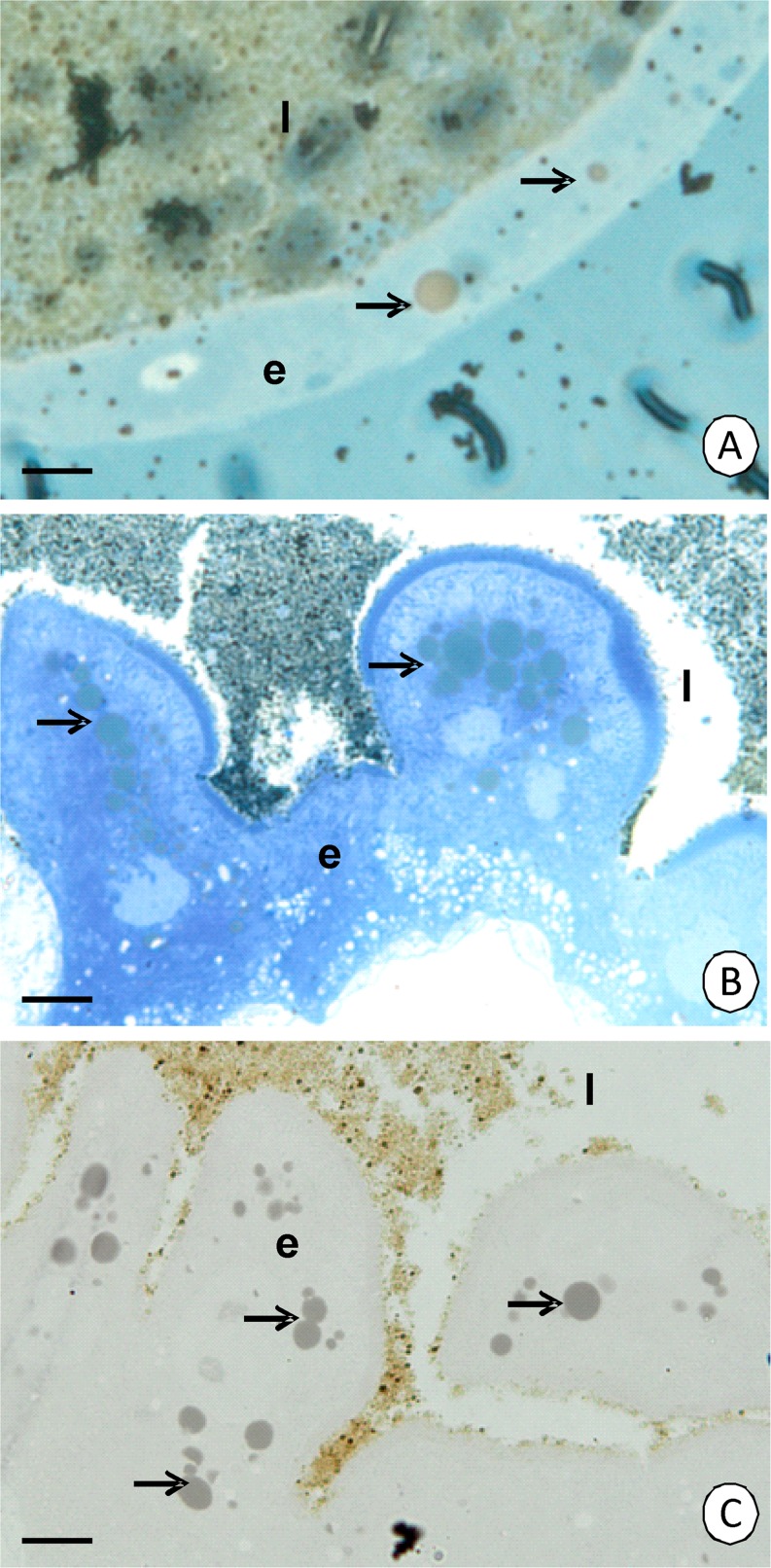
Histochemical staining of the MMG in *Cimex lectularius*. **A** Sudan black B—positive reaction. Light microscopy. *Bar* = 3.5 μm. **B** BPB—positive reaction. Light microscopy. *Bar* = 2.5 μm. **C** *PAS*—negative reaction. Light microscopy. *Bar* = 2.5 μm. MMG lumen (*l*), MMG epithelium (*e*), reserve material (*arrows*).

Histochemical methods have showed that the reserve material accumulated in MMG of both species consists of lipids and proteins, while polysaccharides are absent (Fig 6A–C).

**Fig 7:**
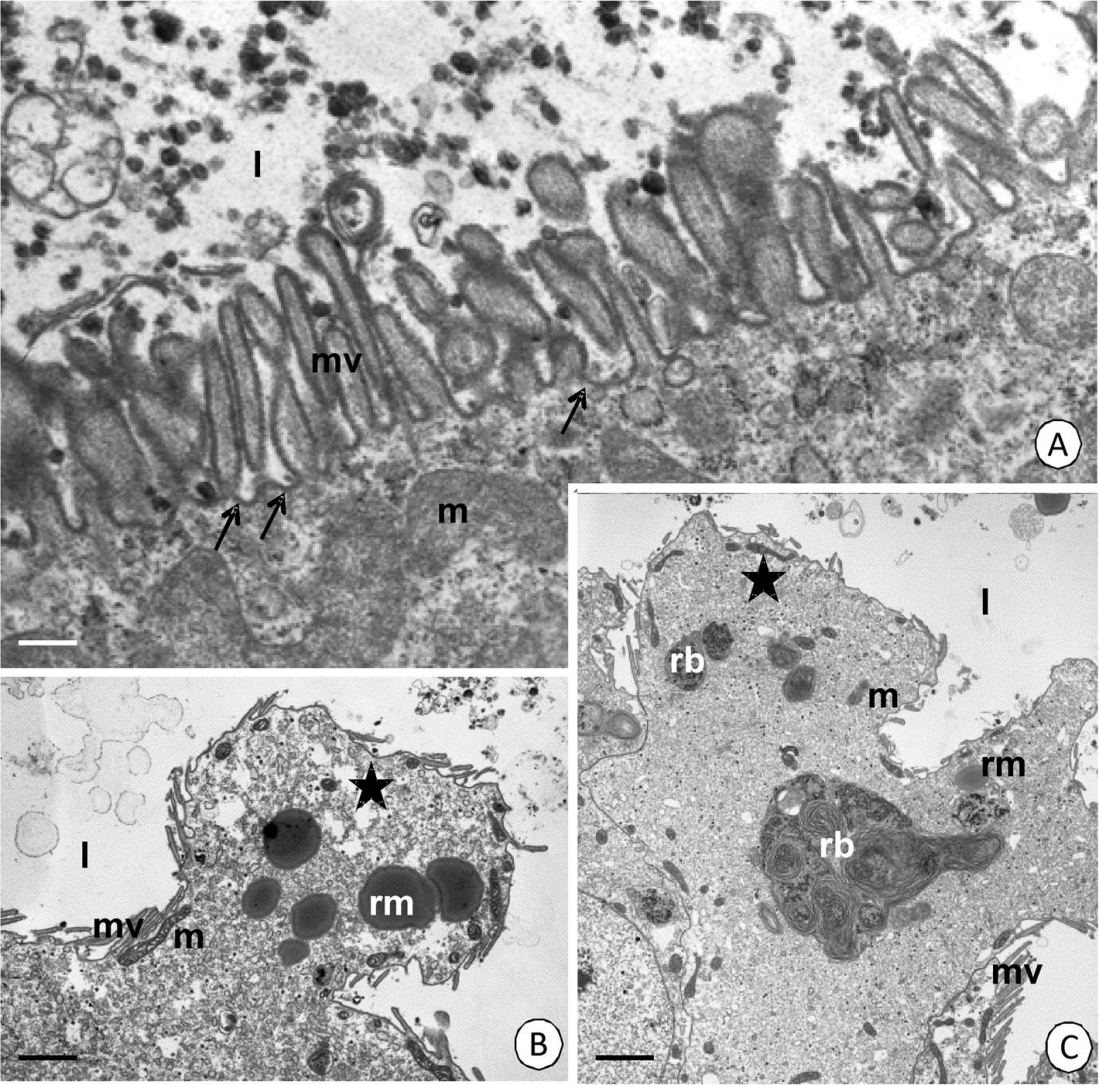
Secretion in AMG and MMG. **A** *Cimex lectularius*. AMG. TEM. *Bar* = 0.1 μm. **B** *Cimex pipistrelli*. AMG. TEM. *Bar* = 1 μm. **C** *Cimex pipistrelli*. MMG. TEM. *Bar* = 0.6 μm. Mitochondria (*m*), microvilli (*mv*), midgut lumen (*l*), reserve material (*rm*), residual bodies (*rb*), the protrusions of the apical cell membrane (*stars*), merocrine vesicles (*arrows*).

The digestive cells secrete substances, which is dependent on merocrine (Fig 7A) and apocrine (Fig 7B, C) secretion. During merocrine secretion, numerous small vesicles with an electron-lucent content adhere to the apical cell membrane and release their content into the midgut lumen (Fig 7A). During apocrine secretion, the apical cell membranes of the digestive cells lose their microvilli and gradually form the protrusion that enters the midgut lumen (Fig 7B, C). However, the secretion occurs only in the AMG and MMG of the midgut, while the cells of the PMG show no secretive abilities.

### Secretory cells

**Fig 8:**
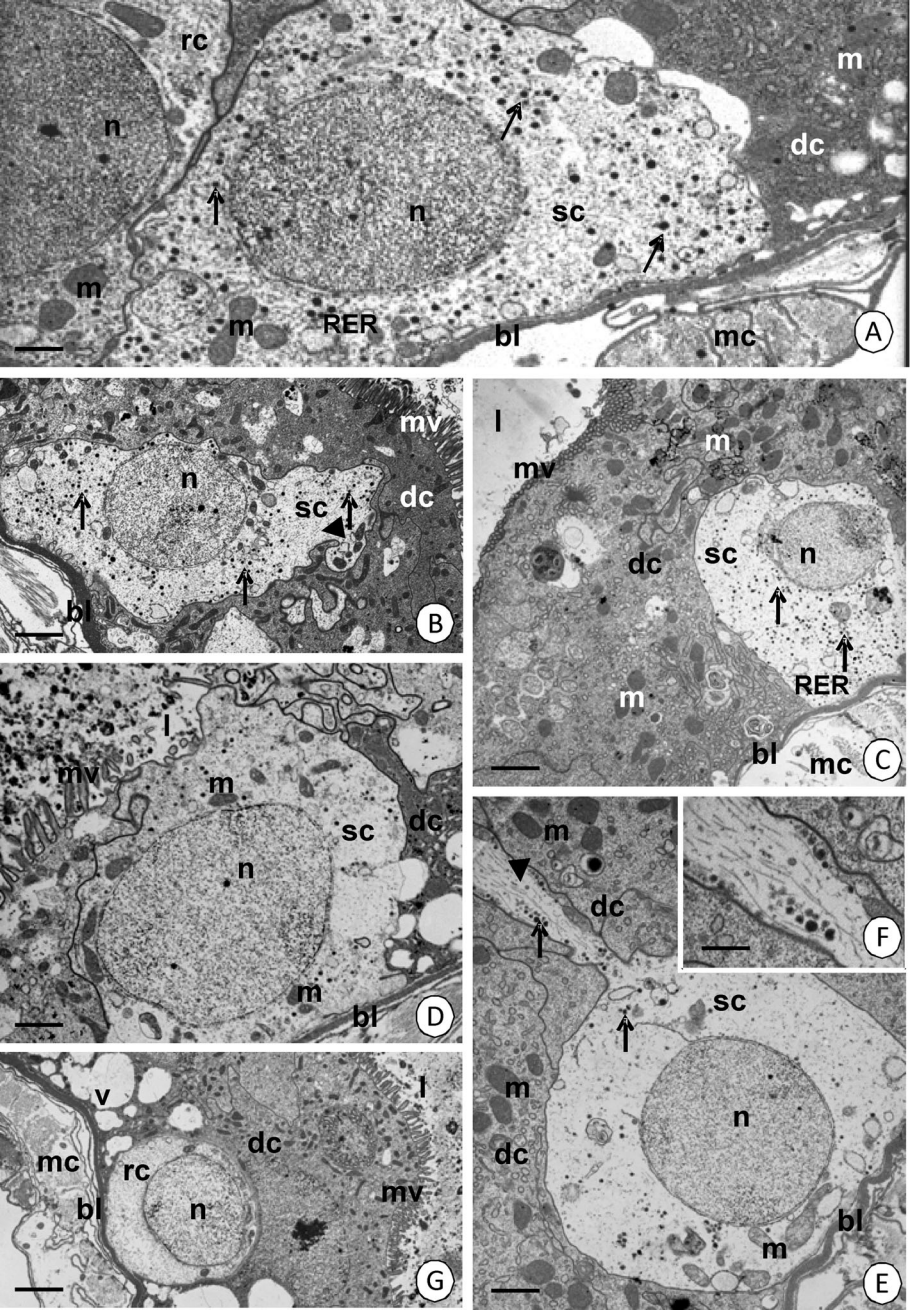
Secretory and regenerative cells. **A**–**F** Secretory cells in the midgut epithelium. **A** *Cimex lectularius*. TEM. *Bar* = 0.4 μm. **B** *Cimex lectularius*. TEM. *Bar* = 1.3 μm. **C** *Cimex pipistrelli*. TEM. *Bar* = 1.8 μm. **D** *Cimex lectularius*. TEM. *Bar* = 0.7 μm. **E** *Cimex pipistrelli*. TEM. *Bar* = 1 μm. **F** *Cimex pipistrelli*. The higher magnification of **F**. TEM. *Bar* = 0.2 μm. Secretory cells (*sc*), regenerative cells (*rc*), digestive cells (*dc*), mitochondria (*m*), nucleus (*n*), microvilli (*mv*), basal lamina (*bl*), midgut lumen (*l*), cisterns of the rough endoplasmic reticulum (*RER*), vacuoles (*v*), electron-dense granules (*arrows*), cytoskeleton (*arrowhead*), visceral muscles (*mc*). **G** Regenerative cell in *Cimex pipistrelli*. TEM. *Bar* = 0.9 μm.

Secretory cells are distributed along the entire length of the intestine. Numerous electron-dense granules can be distinguished in both of the species examined (Fig 8A–F). The cytoplasm has some mitochondria and cisterns of the rough endoplasmic reticulum. The nucleus is electron lucent without distinct patches of heterochromatin (Fig 8A–F). In *C. lectularius*, the secretory cell elongates toward the midgut lumen (Fig 8B). In this species, some mitochondria migrate to form small groups in the apical cytoplasm of the elongated cell, while the remaining organelles are still located in the entire cytoplasm (Fig 8D). However, in *C. pipistrelli* the cell forms a long protrusion toward the midgut lumen with distinct intermediate filaments (about 10 nm in a diameter) and several electron-dense granules (Fig 8E, F). Mitochondria do not form groups (Fig 8E) as they do in *C. lectularius*. The apical cell membrane forms microvilli in both species (Fig 8D).

### Regenerative cells

The regenerative cells are situated along the entire midgut in both of the species examined. They are distributed individually among the basal regions of the columnar cells, and thus, they do not form any nests or crypts. The electron-lucent cytoplasm of the regenerative cells is poor in organelles. Mitochondria and cisterns of the rough endoplasmic reticulum are sporadically distributed in the cytoplasm of both species (Fig 8G). No mitotic divisions and the differentiation of regenerative cells were observed.

## Discussion

The midgut (intestine) is the part of the digestive system that is responsible for the synthesis and secretion of digestive enzymes and the absorption of the nutrients. Therefore, it can be differentiated in some distinct regions with numerous caeca (Rost-Roszkowska 2008). In the majority of Hemiptera that have various feeding strategies, including hematophagous ones, the midgut is divided into three regions—the anterior, middle, and posterior midgut (Silva & Terra 1994, Silva *et al* 1995, Guedes *et al* 2007, Habibi *et al* 2008, Azevedo *et al* 2009, Bandani *et al* 2010, Megiud et al. 2013, Suicmez & Ozmen 2014). The hemipteran midgut has also been described as having four (Nava-Gervasio *et al* 2007) or two regions (Jarial 2005). Miyamoto (1961) and Forattini (1990) incorrectly described the midgut of *C. lectularius* as being composed of only two parts: an anterior and a posterior. However, our studies revealed that the organization of the midgut in *C. pipistrelli* and *C. lectularius* was similar to the one that has commonly been described in the majority of the results mentioned above; it is formed by three regions—the anterior, middle, and posterior midgut. However, their lengths are slightly different. The length of the AMG is identical in both species (25%), the MMG is the longest part in *C. lectularius* (50%), and the PMG is the longest part in *C. pipistrelli* (50%). Both *Cimex* species can feed on a non-natal host, *C. lectularius* from humans on bats, *C. pipistrelli* on humans, but never naturally, and they have a high mortality and do not reproduce (e.g., Wawrocka & Bartonička 2013). Human and bat bloods differ in their chemical composition that is combined with the type of food they eat: human is omnivorous, while bat is insectivorous. There are mainly polysaccharides, saccharides, and lipids in humans food and consequently in humans blood. Therefore, *C. lectularius* needs more complicated digestion than *C. pipistrelli:* bats’ blood is rich in proteins, so the digestion is easier. Hence, we suggest that the midgut of *C. lectularius* is more developed in this species. Cimicidae are temporary ectoparasites, which means that they cannot obtain food continuously. Due to their periodic feeding and then the digestion of a large amount of food, they developed specific adaptation in their midgut. They feed on blood every 5 to 10 min to repletion and then digest the food for about 5–6 days. They are stimulated to search for a blood meal at 3–7-day intervals (Usinger 1966). During this time, alterations in the ultrastructure of the midgut epithelium appear, as was published for the species *Cimex hemipterus Fabricius*, another species that is parasitic to humans (Azevedo *et al* 2009). Moreover, blood is more concentrated than plant sap, the supposed original food of the heteropteran ancestor, and therefore, changes in the midgut structure exist (Goodchild 1966).

The different ultrastructures of the AMG, MMG, and PMG in both of the species examined suggest that these regions must fulfill different functions in the digestive system. When there is no food in the environment, the cytoplasm of the digestive cells in the AMG and MMG is riched in cisterns of the rough and smooth endoplasmic reticulum. The large accumulations of cisterns of the smooth endoplasmic reticulum suggest that the digestive cells of this region are mainly involved in the synthesis of proteins and lipids. However, when blood is the food, the number of fibrillar structures formed by cisterns of the smooth endoplasmic reticulum in the MMG decreases until they completely disappear. The cytoplasm of the digestive cells of the AMG and MMG still remains rich in cisterns of the rough endoplasmic reticulum. Additionally, secretion (both, merocrine and apocrine) starts, which suggests that synthesized enzymes (due to the presence of cisterns of the rough endoplasmic reticulum) are secreted into the midgut lumen. Extracellular digestion occurs. However, when the animal starts to feed on blood, the number of autophagosomes, autolysosomes, and residual bodies increases, which suggests that the autophagy is intensive. Therefore, we can conclude that the process of intracellular digestion, which is connected with the autophagy, also takes place in the AMG and MMG. Additionally, because the AMG is a sack-like structure, it is responsible for the storage of blood, while the cytoplasm of the digestive cells of the MMG accumulates the reserve material, which can be exploited by the animal during periods of starvation. In addition, the accumulation of large vacuoles, which grow in size after blood feeding and during blood digestion, suggests that the AMG and MMG also take part in water absorption from ingested food. Similar functions of the three distinct regions of the midgut have been also described for some other hematophagous and non-hematophagous hemipterans (Dow 1987, Billingsley 1990, Terra 1990, Silva *et al* 1995, 1996, Azevedo *et al* 2009, Fialho *et al* 2013, Megiud et al. 2013). In *R. prolixus*, the crop (AMG) is responsible for water transport, ion regulation, and the accumulation of lipids and glycogen. Enzymes are mainly produced and secreted in the anterior intestine, while the posterior intestine is the place where only sporadic secretory activity occurs, although this region plays an important role in the absorption of digested nutrients and the storage of carbohydrates (Billingsley 1988, Terra 1988, 1990). In *C. hemipterus*, water absorption, ion regulation, digestion, and the storage of lipids and polysaccharides occur in the anterior midgut. The middle midgut is connected only with digestive processes in this species, while the posterior midgut fulfills a role in nutrient absorption and hemoglobin digestion (Azevedo *et al* 2009). The zoophytophagous hemipteran predator, *Podisus nigrispinus Stal*, feeds on prey and plant xylem content (Torres *et al* 2010), and its midgut is composed of three regions that differ in its ultrastructure and functions (Fialho *et al* 2013). In the PMG of both *Cimex* species, the reserve material and vacuoles do not appear after feeding with blood and no secretion was been observed. However, the increasing number of spherites suggests a role of the PMG in the accumulation of toxic substances that can originate from food.

During digestion, blood generates many toxic particles (e.g., reactive oxygen) (Dunkov *et al* 2002, Taketani 2005), which create different mechanisms for their neutralization (Okuda *et al* 2005, 2007, Graça-Souza *et al* 2006). In hematophagous invertebrates (e.g., Anoplura, Psocodea), the midgut epithelium has numerous electron-dense structures called hemoxisomes (Silva *et al* 2006) or spherocrystals (Azevedo *et al* 2009). These are membranous vesicles that have electron-dense material and are surrounded by cisterns of the rough endoplasmic reticulum. They are responsible for the detoxification of intracellular heme (Silva *et al* 2006). Similar structures are present in the epithelial cells of the digestive system in hematophagous arachnids, and they are suspected to be responsible for the formation of endosomes and the digestion of blood (Tarnowki & Coons 1989, Filimonova 2008). Similar granules have been described in the cytoplasm of the AMG and MMG in both *Cimex* species after blood entered the midgut lumen, and they are surrounded by some cisterns of the rough endoplasmic reticulum. However, due to the fact that intracellular digestion occurs in both of these intestine regions, we can conclude that such structures take part in the digestion of blood.

Secretory cells of the intestine (midgut) epithelium of invertebrates are described as goblet-shaped glandular cells scattered between the digestive cells. They can be found either as cells of the closed type, which do not contact the midgut lumen or as open type cells, whose apical cell membrane reaches the midgut lumen. The most characteristic feature of secretory cells is the presence of abundant granules that are of a different electron density (Endo & Nishiitstsuji-Uwo 1981, Punin *et al* 2000, Neves *et al* 2003, Rocha *et al* 2014). The appearance of cisterns of the rough and smooth endoplasmic reticulum in the cytoplasm of secretory cells is connected with intensive synthesis of the contents of the electron-dense and electron-lucent granules. These cells have been described as the endocrine cells because they synthesize numerous hormones—they are, e.g., FRMF-amide-, serotonin-, calcitonine-, cholecystokinin-, pancreatic polypeptide-, and neurotensin-positive (Montuenga *et al* 1989, Punin *et al* 2000, Neves *et al* 2002). The secretory cells described in both species of *Cimex* correspond to open-typed secretory cells because they reach the midgut lumen. Only one small difference occurred between two species analyzed—the cell is elongated toward the midgut lumen with distinct patches of accumulated mitochondria (*C. lectularius*), or the cell formed a long protrusion toward the midgut lumen and mitochondria did not accumulate (*C. pipistrelli*). However, the different shape of such cells is not surprising, as many types of secretory cells have been discovered in invertebrates (Punin *et al* 2000). To state that these cells fulfill the role the endocrine cells, some additional histochemical staining should be performed.

The regenerative cells in the midgut epithelium of insects, due to which the process of its renewal can proceed, might form groups that are called regenerative nests or crypts, or they might appear as single cells arranged between the basal regions of the epithelial cells. The regenerative cells in Hemiptera may also form regenerative nests (Fialho *et al* 2013, Teixeira *et al* 2013). Because the regenerative cells are able to proliferate and differentiate, they can also be treated as midgut stem cells (Cruz-Landim *et al* 1996, Rost *et al* 2005, Rost 2006). The regenerative cells of both *Cimex* species have the cytoplasm that is poor in organelles. They are distributed individually among the basal regions of the digestive cells. However, their ultrastructure is characteristic for other hematophagous and non-hematophagous Hemiptera (Billingsley 1990, Azevedo *et al* 2009, Fialho *et al* 2013, Teixeira *et al* 2013). Regenerative cells proliferation and differentiation have not been observed in both species examined. It is high probably that as in other insects, the proliferation and cells differentiation occur after the beginning of cells degeneration (Rost *et al* 2005, Rost-Roszkowska 2008, Sosinka *et al* 2014). However, this should be studied in more detail.

The results of our studies confirm the suggestion of former authors (Goodchild 1966, Terra 1988, 1990, Schumacker et al. 1993), who concluded that a conserved digestive pattern appears in Hemiptera that ingest various kinds of food (fungus-feeding, detritus-feeding, seed-feeding, predaceous, zoophytophagous, phytophagous and hematophagous) and that it is not connected with insects’ diet but is correlated with phylogeny. We agree with Fialho *et al* (2009, 2012, 2013) that the structure of the digestive tract of insects is not attributed solely to diet but to the basic adaptation of an ancestor.

We conclude that (1) the AMG fulfills the role of storing food and synthesizing and secreting enzymes; (2) the MMG is the main organ for the synthesis of enzymes, secretion, and the storage of the reserve material; (3) the AMG and MMG take part in water absorption; (4) the PMG is the organ in which spherites accumulate and (5) the morphology and ultrastructure of the digestive epithelium have a conserved pattern in hematophagous as well as in non-hematophagous Hemiptera.
